# Deletion of *Fgf14* confers resilience to basal and stress-induced depressive-like behavior and reduces anxiety in mice

**DOI:** 10.1038/s41398-025-03361-z

**Published:** 2025-04-09

**Authors:** Francesca Montarolo, Anita Maria Rominto, Luna Berrino, Antonio Bertolotto, Fernanda Laezza, Filippo Tempia, Eriola Hoxha

**Affiliations:** 1 Department of Neurosciences “Rita Levi Montalcini”, University of Turin, Turin, Italy; 2 Neuroscience Institute Cavalieri Ottolenghi (NICO), Orbassano, TO Italy; 3 Department of Pharmacology and Toxicology, University of Texas Medical Branch, Galveston, TX USA

**Keywords:** Neuroscience, Diseases

## Abstract

Depression is a mental illness characterized by despair behavior, inability to feel pleasure, and social withdrawal. Causes are not yet clarified, but stress is a condition that induces depression. Neuronal alterations, comprising maladaptive neuronal plasticity and excitability, are present in both responses to stress and depression. Fibroblast growth factor 14 (*Fgf14*) controls neuronal excitability and proper action potential firing by stabilizing voltage-dependent sodium (Na_v_) channels into the axon. *Fgf14*-Na_v_ channels complex is regulated by glycogen synthase kinase 3. Recently, *Fgf14* has been genetically associated to depression. However, little is known about its role in controlling stress-induced depression. This study demonstrates that female *Fgf14*^*−/−*^ mice are resilient to depression, as reported by reduced level of despair behavior, *anhedonia*, and increased sociability. Also, a reduction of anxious-like behavior was highlighted. *Fgf14*^*−/−*^ mice showed increased expression of cannabinoid receptor without alterations of dopaminergic system in mPFC, suggesting a link between *Fgf14* and endocannabinoid system in the control mechanisms underlying depression. Neuronal activity was assessed by analyzing cFOS expression during basal and following acute stress induced by tail suspension test (TST). The analysis revealed that neuronal activation in mPFC and VTA was correlated to immobility, where ratio of cFOS expression over immobility was significantly higher in *Fgf14*^*−/−*^ mice. This suggests that higher neuronal activity might be involved in resilience to depression. In resilient *Fgf14*^*−/−*^ mice, TST-induced acute stress caused activation only in pyramidal neurons. Our findings suggest that *Fgf14* is involved in stress-coping mechanisms and could be targeted to improve resilience to depression.

## Introduction

Depression is a common mental disorder characterized by despair behavior, inability to feel pleasure and social withdrawal. It affects about 5% of the population worldwide constituting a major global burden of disease [1]. In human subjects and experimental animals, depression is associated with multiple neural alterations, including a decreased volume of medial prefrontal cortical (mPFC) areas, like subgenual and cingulate cortex [2–4]. Stress is the main condition that can induce depression in susceptible individuals. Actually, the responses to stress and depression share multiple neural alterations [4–6]. The brain circuit involved in the response to stress and in depression includes ventral hippocampus, ventral tegmental area (VTA), nucleus accumbens (NAc), amygdala, lateral habenula [6, 7]. However, very little is known about the molecular mechanisms underlying the response to stress and depression. Endocannabinoids and monoaminergic systems have been implied, but their mechanistic role in depression is far from clear [8, 9]. For this reason, the current pharmacological treatments for depression, including selective serotonin reuptake inhibitors (SSRIs), have a low efficacy and delayed effects [10–13]. Some alterations in neuronal activity associated with stress and depression have been reported [6, 7]. These include maladaptive neuronal plasticity and excitability. Notably, in neurons the most significant cellular domain, able to influence neuronal plasticity and excitability, is the axon initial segment (AIS), which is characterized by a high density of voltage-gated sodium channels (Na_v_) [14, 15]. In this context, the fibroblast growth factor 14 (*Fgf14*) which is a non-secreted intracellular accessory protein of voltage-dependent sodium channels Na_v_1.1, Na_v_1.2 and Na_v_1.6 [16, 17] has a crucial importance. By isoform-specific binding to the C-terminal domain of Na_v_ channels, *Fgf14* controls their localization to the AIS to preserves action potential firing [17–19]. Moreover, *Fgf14* modulates the gating properties of Na_v_ channels, necessary for proper neuronal excitability maintenance [17–20]. *Fgf14* is expressed in various regions of the central nervous system including the cerebellum, cerebral cortex, hypothalamus, basal ganglia, amygdala, hippocampal formation and midbrain [21]. This expression suggests that *Fgf14* modulates neuronal function in these regions, thus controlling multiple cognitive and emotional responses.

Indeed, mutations in the *Fgf14* gene were first associated with the spino-cerebellar ataxia type 27 (SCA27), a naturally occurring neurodegenerative disorder characterized by ataxia and deficits in cognition, and memory [22–25]. *Fgf14*^*−/−*^ mice have motor deficits including an ataxic gait with shuffling footprint patterns, a widened stance, lack of forefoot-hindfoot correspondence and a short latency to fall in the accelerated rotarod test [26, 27]. However, *Fgf14*^*−/−*^ and control mice were not found to differ in spontaneous locomotor activity [26]. Moreover, male mice with genetic ablation of *Fgf14* (*Fgf14*^*−/−*^ mice) show a marked reduction of aggressive and sexual behavior in addition to the ataxic phenotype [28, 29]. Recently, *FGF14* has been reported by linkage and genome wide association studies (GWAS) as a putative risk factor for neuropsychiatric diseases including major depressive disorder [30], and bipolar disorder [31, 32].

It is interesting to note that the *Fgf14* binding to Na_v_ channels is regulated by phosphorylation by kinases, [33, 34] including the glycogen synthase kinase 3 (GSK3) [35], which is a key enzyme linked to major depressive disorder and bipolar disorder [35–38]. Specifically, increased GSK3 activity is associated with susceptibility to mood disorders [38]. This fact raises the hypothesis that a possible pathway associated with susceptibility to depressive disorders is based on a GSK3-dependent high activation of *Fgf14*. For this reason, *Fgf14*^*−/−*^ mice might represent a model for resilience to depression. This study aims to identify resilience to stress and depression in female *Fgf14*^*−/−*^ mice and to assess the mechanism of such resilience by analyzing changes in neuronal activity.

## Materials and methods

### Animals

Two to four months old female *Fgf14*^*−/−*^ mice and their wild type (WT) littermates (strain C57/Bl6) in estrous phase were used for all experimental paradigms. We chose females for this study about depression to reduce the variability due to possible gender differences and because in humans the prevalence of depression is higher in women compared to men [39]. The *Fgf14*^*−/−*^ mice were obtained from Prof. Fernanda Laezza, University of Texas Medical Branch USA. Adequate measures were taken to minimize pain and discomfort. We included in the study female animals with no signs of illness or physical abnormalities. Were excluded from the study animals that showed abnormal behavior, signs of injury, or illness during the acclimation period or prior to the start of the experiment. No animals were randomized in the analyses. All the experimental procedures were carried out at Neuroscience Institute Cavalieri Ottolenghi (NICO) and approved by the Ethical Committee of the University of Torino and authorized by the Italian Ministry of Health (authorization number: 822/2016-PR). Experiments have been carried out in accordance with the European Communities Parliament and Council Directives of 24 November 1986 (86/609/EEC) and 22 September 2010 (2010/63/EU).

### Behavioral tests

Mice underwent the behavioral tests always during the light phase of the cycle. The experiments were performed under dim white light conditions (2 lux). At the end of each trial, the equipment was accurately cleaned with ethanol 2% and water. Where needed (i.e. tail suspension test; TST, elevated plus maze test; EPM, open field test; OF, and three chamber sociability test), behavioral procedures were video-recorded and scored by an individual blind to the genotype of the mouse. Data obtained from EPM, OF and three chamber sociability tests were analyzed using Ethovision XT15 video track system (Noldus Information Technology, Wageningen, The Netherlands).

#### Tail suspension test (TST)

The animals (*n* = 7 WT; *n* = 6 *Fgf14*^*−/−*^) were transported to the testing room and left undisturbed for at least 45 min before the test. The test was performed as previously described [40]. The suspension box (55 × 45, height 33 cm) is composed of two compartments separated by a wall, to prevent animals from observing or interacting each other during the test. Each mouse was suspended by its tail using a piece of tape (17 cm) able to securely adhere to both the mouse’s tail and the suspension bar positioned at the top of the box. Before the application of the tape, a clear hollow cylinder (4 cm length, 1.2 cm outside diameter, 3 grams) was placed around the tail of mouse to prevent mouse to climb its tail during the test. The tests were video-recorded for 6 min. Time of immobility and the latency to first immobility were measured. Small movements that were confined to the front legs but without the involvement of the hind legs were counted as immobility. To have the unstressed control groups for histological procedures, a set of mice (*n* = 7 WT; *n* = 4 *Fgf14*^*−/−*^) were transported to the testing room throughout the experiment but were not tested for TST.

#### Sucrose preference test

Mice (*n* = 13 WT; *n* = 8 *Fgf14*^*−/−*^) were individually placed in a cage (35 × 20, height 20 cm) for at least 3 days prior to introduction of two water bottles sucrose preference test. Animals were allowed to acclimate to two bottles availability for at least 4 days prior to start of testing. Bottles were swapped daily to minimize potential side bias. After the period of habituation, one bottle was filled with a premeasured amount of water and the other one was filled with a premeasured amount of 2% sucrose. This configuration was left for four days, rotating the bottle position daily. Sucrose consumption was measured as ratio between sucrose consumed per day and sucrose plus water consumed per day.

#### Three-chambered sociability test

Mice (*n* = 9 WT; *n* = 9 *Fgf14*^*−/−*^) were individually housed (cage: 30 × 15, height 13 cm) the day before performing the test. Animals were transported to the testing room and left undisturbed for at least 45 min before the test. The test, using a custom-made clear Plexiglas box (17.5 × 41.5, height 22 cm) partitioned into three chambers of equal size, was performed as previously described [28]. The test was divided in two phases, such as habituation, and social behavior. During habituation, the mouse was confined in the middle chamber for 10 min. After this phase the doors were opened, and the mice was permitted to move freely in all chambers for other 10 min. During social behavior, the test mouse was confined again in the middle chamber. An inverted empty wire cup-cage (object, wire pencil cup, Galaxy Cup, Kitchen Plus, http://www.kitchen-plus.com) and a wire cup containing a stranger mouse (mouse) were placed into left and right chambers. The doors were re-opened, and the mouse was allowed to explore all chambers for 10 min. The comparison of the time spent with mouse versus object indicated the sociability of animals. Stranger mouse was housed in the same facility but had no prior contact with test mice. It was trained for two sessions of 15 min the day before the test. The observer remained in the room and only mice who at the end of 15 min do not grip to the wire cup-cage were chosen.

#### Elevated plus maze test (EPM)

Mice (*n* = 11 WT; *n* = 9 *Fgf14*^*−/−*^) were individually housed (cage: 30 × 15, height 13 cm) the day before performing the test. Animals were transported to the testing room and left undisturbed for at least 45 min before the test. The test was performed as previously described [41]. The EPM test apparatus was plus cross shaped constructed from grey forex raised 60 cm above the floor. It comprised two open arms (30 × 5 × 0.20 cm) and two closed arms (30 × 5 × 15 cm walls) originating from a central platform (5 × 5 cm). At the beginning of each trial each animal was gently placed in the center of the plus maze, facing an open arm and it was allowed to explore the maze for 5 min. The number of entries in either open arm, their respective cumulative time spent, and the distance travelled in the maze were reported. An animal was considered to have entered an arm of the plus maze when all four paws had left the central platform.

#### Open field test (OF)

Mice (*n* = 7 WT; *n* = 10 *Fgf14*^*−/−*^) were individually housed (cage: 30 × 15, height 13 cm) the day before performing the test. The animals were transported to the testing room and left undisturbed for at least 45 min before the test. The test was performed as previously described [41–43]. Each animal was placed in the corner of the arena (50 × 50, height 50 cm) for 1 h. Total and 10 min bins distance travelled in the arena and in the central area (25 × 25 cm) were reported.

### Biomolecular analysis

Mice (*n* = 7 WT; *n* = 8 *Fgf14*^*−/−*^) were euthanized by inhalation of isoflurane and brains were removed. Medial prefrontal cortices (mPFC, Bregma 1.98 mm, Interaural 5.78; Bregma 1.34 mm, Interaural 5.14) nucleus accumbens (NAc), caudate putamen (CPu, Bregma 1.54 mm, Interaural 5.34; Bregma 0.86 mm, Interaural 4.66), and amygdala (Bregma −1.58 mm, Interaural 2.22; Bregma −2.30 mm, Interaural 1.50) were manually dissected from 300 μm-thick coronal sections cutter by vibratome (Leica Microsystems GmbH, Wetzlar, Germany). All samples were rapidly frozen in 2-methylbutane in dry ice and total RNA was isolated by extraction with the Pure Link RNA Mini Kit (Thermo Fisher Scientific, US) according to the manufacturer’s instructions. Total RNA was reverse transcribed to complementary DNA (cDNA) at a final concentration of 20 ng/μL using the High-Capacity Kit (Thermos Fisher Scientific, Waltham, MA, USA). Gene expression analysis was performed by real-time PCR using Applied Biosystems’ TaqMan Assay-on-demand-TM gene expression products (Thermos Fisher Scientific, Waltham, MA, USA). Transcriptional expression was normalized using phosphoglycerate kinase 1 (PGKI) as reference gene. Expression levels of target genes were calculated by the normalized comparative cycle threshold (Ct) method (2^-^ΔCt). Protein analysis was performed as previously described [44]. Briefly, mPFC from WT (*n* = 7) and *Fgf14*^*−/−*^ (*n* = 6) mice were resuspended and lysed in 20% (w/v) RIPA buffer (25 mM Tris-HCl pH 7.4, 150 mM NaCl, 1 mM EGTA, 1 mM EDTA, 1 mM dithiothreitol, 0.5 mM PMSF, 10 μg/mL Aprotinine, 10 μg/mL Leupeptine, 2 mM sodium orthovanadate). The lysates were centrifuged at 10,000 g for 20 min at 4 °C. Twenty micrograms of proteins were separated by using a 4–12% Bis-Tris precast gel (Life Technologies) and transferred onto nitrocellulose membrane using an iBlotTM dry blotting system (Life Technologies). Membranes were than blocked with EveryBlot Blocking Buffer (Bio-Rad) and then incubated overnight at 4 °C with primary antibodies to CB1 (1:2000, ProteinTech, Cat# 17978-1-AP, RRID: AB_10859098) and Gapdh (1:1000, Abcam, Cat# ab181602, RRID: AB_2630358). HRP-conjugated goat anti-rabbit (1:5000, Bio-Rad, 170-6515, RRID: AB_11125142). For detection we used Luminata Forte Western substrate (WBLUF0100, Merck-Millipore, Darmstadt, Germany). Images were acquired by Chemidoc (Bio‐Rad) and quantified by ImageLab software (RRID: SCR_014210, Bio‐Rad). Densitometric values were normalized to Gapdh. The protein extracts were run at least three times to check reproducibility.

### Histological procedures and image analysis

At the end of TST procedure, mice (*n* = 7 unstressed WT; *n* = 7 TST-induced acute stress WT; *n* = 4 unstressed *Fgf14*^*−/−*^*; n* = 6 TST-induced acute stress *Fgf14*^*−/−*^) were left undisturbed for 2 h in the testing room, then they were deeply anesthetized (ketamine 200 mg/kg, xylazine 50 mg/ kg) and transcardially perfused with 4% paraformaldehyde in 0.12 M phosphate buffer, pH 7.2–7.4. The brains were removed and immersed in the same fixative at 4 °C for 2 h and then cryo-protected in 30% sucrose in 0.12 M phosphate buffer. Brains were frozen and serially cut by a cryostat in 30 μm-thick coronal sections collected in phosphate buffered saline (PBS). To detect cFOS positivity, slices were incubated overnight at 4 °C with the polyclonal anti-rabbit cFOS (9F6, Cell Signaling Technology, MA, USA, Cat# 2250, RRID: AB_2247211) diluted 1:1000 in PBS with 0.25% Triton ×-100 and 1.5% normal donkey serum. Immunohistochemical reactions were performed by the avidin–biotin–peroxidase method (Vectastain ABC Elite kit; Vector Laboratories, Burlingame, CA, USA) and revealed using 3,3′-diaminobenzidine (3% in Tris–HCl) as chromogen. Images were acquired by means of the ZEISS Axioscan 7 (Oberkochen, Germany) using the 20X magnification.

To detect cFOS positivity in GAD67 positive (^+^) GABAergic neurons, slices were incubated overnight at 4 °C with the polyclonal anti-rabbit cFOS (9F6, Cell Signaling Technology, MA, USA) and with the monoclonal anti-mouse GAD67 (Merck-Millipore, Cat# MAB5406, RRID: AB_2278725) diluted (1:1000) in PBS with 0.25% Triton ×-100 and 1.5% normal donkey serum. Immunofluorescent reactions were revealed by secondary antibodies CY3 donkey anti-rabbit IgG (H+L) (Cat# 711-165-152, RRID: AB_2307443, Jackson ImmunoResearch, Europe LTD), and AlexaFluor647 donkey anti-mouse IgG (H+L), (Cat# 715-605-151, RRID: AB_2340863, Jackson ImmunoResearch). 4,6-diamidino-2-phenylindole (DAPI, Fluka, Saint Louis, USA) was used to counterstain cell nuclei. Image stacks with 0.5 μm-thick planes using the 40× magnification. were acquired by means of the Leica TCS SP5 confocal microscope. The number of cFOS positive (^+^) cells/mm^2^ (cell density) in immunohistochemical images and the density of cFOS^+^GAD67^+^ and cFOS^+^GAD67^-^ cells among total nuclei in immunofluorescent images were manually evaluated by means of the ImageJ software (http://rsbweb.nih.gov/ij/index.html) using the cell counter plugin.

Accordingly to the Mouse Brain in Stereotaxic coordinates (Paxinos G. and Franklin KB) total mPFC, anterior cingulate area (ACA), prelimbic area (PL), infralimbic area (IL) (Bregma 1.98 mm, Interaural 5.78; Bregma 1.34 mm, Interaural 5.14), nucleus accumbens (NAc) shell, NAc core (Bregma 1.54 mm, Interaural 5.34; Bregma 0.86 mm, Interaural 4.66), amygdala, lateral habenula, dorsal hippocampus (HIP) (Bregma −1.58 mm, Interaural 2.22; Bregma −2.30 mm, Interaural 1.50), ventral HIP, ventral tegmental area (VTA) (Bregma −2.92 mm, Interaural 0.88; Bregma −3.40 mm, Interaural 0.40) were identified and evaluated. At least three sections for animal were evaluated. Image analyses were performed by an experimenter blind to the genotype of the animal.

### Statistical analysis

Statistical analyses were carried out by GraphPad Prism 8 (GraphPad Software Inc., La Jolla, CA, USA) and included Mann-Whitney U test, Kruskal–Wallis test with Dunn’s post hoc test, two-way ANOVA repeated measures with Bonferroni’s multiple comparisons test, and Pearson correlation. The normality of distribution was assessed by the Shapiro-Wilk test, and homogeneity of variance with Levene. Parametric or not-parametric tests were applied, as appropriate. Statistical significance was considered at *p* values < 0.05. To estimate the number of animals to be used to achieve statistical significance, we performed an a priori statistical power analysis. The expected variability for each type of experiment was derived from our previous publications in which we had used the same experimental techniques [28] or from literature data. The number of animals to be used in each experiment was estimated by setting the probability of a type I error (α err prob = 0.05) and power of 0.8 (1-β err prob = 0.8) [45]. Data collection stopped when sample size was reached.

## Results

### Resilience to depressive-like behavior in *Ffg14*^−/−^ mice

**Fig. 1:**
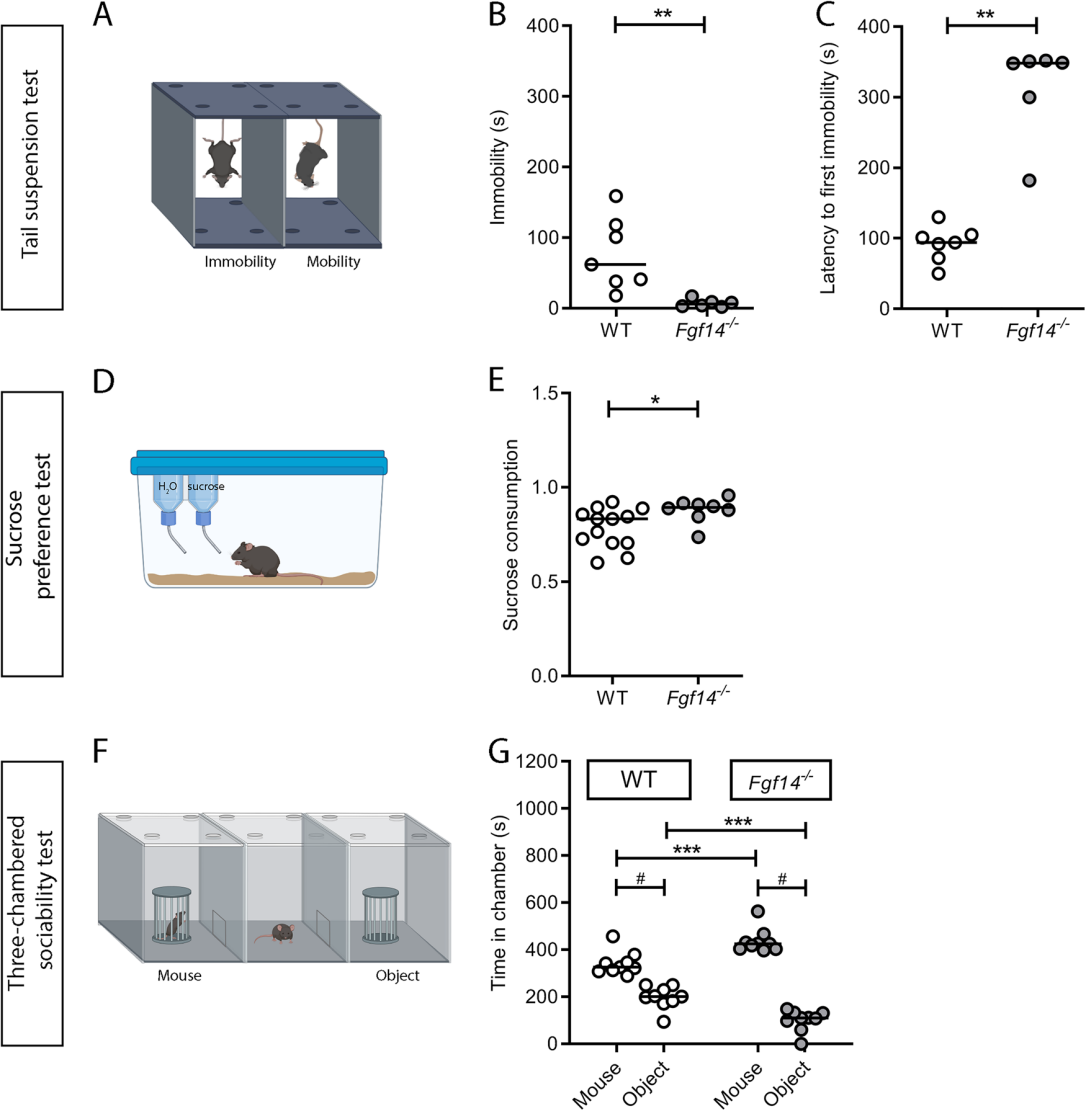
Behavioral tests related to depression in WT and *Ffg14*^*−/−*^ mice. WT (empty circles) and *Fgf14*^*−/−*^ (filled circles) mice were tested in the tail suspension (TST, **A**–**C**), the sucrose preference **D**, **E**, and the three-chambered sociability **F**, **G** tests. **A** Schematic representation of the tail suspension test. *Fgf14*^*−/−*^ mice show a decreased level in immobility time **B**, and an increase in the latency to the first immobility episode **C** compared to WT in the TST, demonstrating a lower level of despair behavior. **D** Schematic representation of the sucrose preference test. *Fgf14*^*−/−*^ mice show an increase in the level of sucrose consumption compared to WT in the sucrose preference test **E**, demonstrating a lower level of *anhedonia*. **F** Schematic representation of three-chambered sociability test. *Fgf14*^*−/−*^ mice show no deficits of interaction with the stranger mouse relative to the object in the three-chambered sociability test. However, *Fgf14*^*−/−*^ mice show an exaggerated interaction in sociability respect to WT **G**. The bars indicate median values. * two-way ANOVA, *p* < 0.05, ^**^ two-way ANOVA, *p* < 0.01, ***two-way ANOVA, *p* < 0.001, ^#^ Mann-Whitney test, *p* < 0.05.

The degree of despair behavior was assessed by TST (Fig. 1A). In the test, the immobility time and the latency to the first immobility differed significantly between genotypes. In particular, *Fgf14*^*−/−*^ mice showed a decreased immobility time (Mann-Whitney U test, **p = 0.001, Fig. 1B) and an increased latency to the first immobility episode (Mann-Whitney U test, **p = 0.001, Fig. 1C) compared to their WT littermates, showing a resilience to stress-induced depressive-like behavior. The inability to feel pleasure (*anhedonia*) was investigated in the sucrose preference test (Fig. 1D). This test highlighted a significant increase in the level of sucrose solution consumption by *Fgf14*^*−/−*^ mice respect to their WT littermates (Mann-Whitney U test, *p = 0.023, Fig. 1E), indicating a higher ability to experience pleasure. Sociability was evaluated by the three-chamber test (Fig. 1F). *Fgf14*^*−/−*^ mice, compared to their WT littermates, showed an exaggerated interaction with the stranger mouse and a reduced interaction with the inanimate object (two-way ANOVA repeated measures Bonferroni’s multiple comparison test, mouse ***p = 0.0005, object ***p = 0.0003, Fig. 1G). As expected, *Fgf14*^*−/−*^ mice showed no deficits in the choice of interaction with the stranger mouse rather than with the inanimate object (Mann-Whitney U test, #*p* < 0.0001, Fig. 1G).

### Reduced anxious-like behavior of *Ffg14*^−/−^ mice

**Fig. 2:**
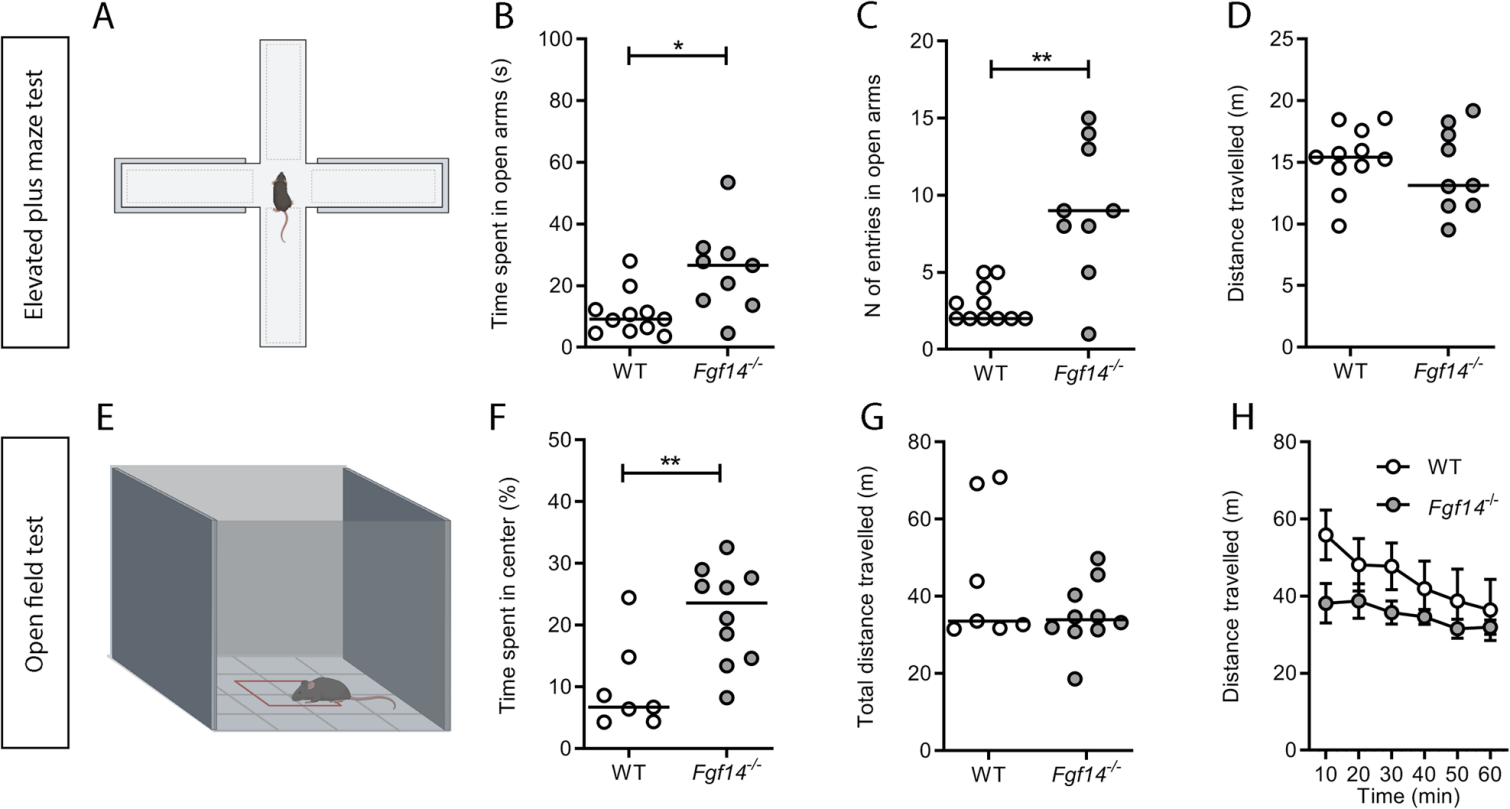
Behavioral tests related to anxiety in WT and *Ffg14*^*−/−*^ mice. WT (empty circles) and *Fgf14*^*−/−*^ (filled circles) mice were tested in the elevated plus maze (EPM) test (**A**–**D**), and the open field (OF) test (**E**–**H**). **A** Schematic representation of the EPM. *Fgf14*^*−/−*^ mice spend more time in open arms **B**, with an increased number of entries in them **C** compared to WT, showing a decreased anxiety-like behavior. Distance travelled is not different between genotypes **D**. **E** Schematic representation of the OF. *Fgf14*^*−/−*^ mice spend more time in the center of arena relative to WT **F**, confirming a decreased anxiety-like behavior. Distance travelled, reported as total distance **G**, or binned every 10 min **H** is not different between genotypes. The bars indicate median values. ^*^p < 0.05, ^**^p < 0.01.

Anxiety is often associated with depression. Therefore, the basal anxious-like behavior was evaluated by EPM and OF tests. The level of anxiety is evaluated considering the time spent in open arms and the number of entries in same arms of EPM (Fig. 2A). The analysis revealed that *Fgf14*^*−/−*^, compared to WT mice, spent more time in open arms (Mann-Whitney U test, *p = 0.012, Fig. 2B) and showed an increased number of entries (Mann-Whitney U test, **p = 0.002, Fig. 2C). No significant difference between genotypes was observed in the distance travelled (Mann-Whitney U test, *p* = 0.603, Fig. 2D). The reduced level of anxiety of *Fgf14*^*−/−*^ mice was also corroborated by the analysis of the percentage of time spent in the center of arena during the OF test (Fig. 2E). In fact, *Fgf14*^*−/−*^ mice spent more time in the center of arena respect to their WT littermates (Mann-Whitney U test, **p = 0.009, Fig. 2F). No significant difference in the distance travelled was found between genotypes, neither reported as total distance (Mann-Whitney U test, *p* = 0.475, Fig. 2G) nor as function of 10 min bins (two-way ANOVA repeated measures F = 1.76, *p* = 0.131, Fig. 2H).

### Alteration of CB1 receptor expression in mPFC of *Ffg14*^−/−^ mice

**Fig. 3:**
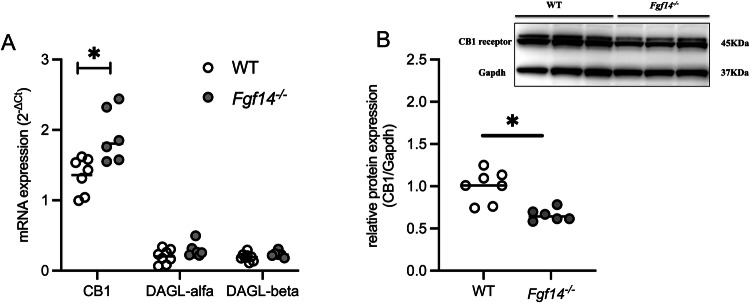
Expression levels of endocannabinoid and dopaminergic systems in the mPFC. Comparison of gene expression levels in the mPFC of WT (empty circles) and *Fgf14*^*−/−*^ (filled circles) mice of cannabinoid receptor 1 (CB1), diacylglycerol lipase alfa (DAGL-alfa) **B**, diacylglycerol lipase beta (DAGL-beta) **A**. *Fgf14*^*−/−*^ mice show an increase in the gene expression level of CB1 respect to WT. **B** (inset) Representative western blots of mPFC extracts from WT and *Fgf14*^*−/−*^ mice. Densitometric quantification shows reduced levels of CB1 receptor protein in the mPFC of *Fgf14*^*−/−*^(filled circles) mice compared to WT littermates (empty circles). Gapdh served as loading control. The bars indicate median values. ^*^p < 0.05.

The mPFC is the main hub of the network involved in the response to stress and in mood disorders [2–4]. In this cerebral region, we analyzed the endocannabinoid and the dopaminergic systems which are involved in the modulation of mood [8, 9]. By semi-quantitative mRNA analysis, *Fgf14*^*−/−*^ mice showed an increase in the level of the CB1 receptor transcripts relative to their WT littermates (Mann-Whitney U test, *p = 0.014, Fig. 3A). No other significant differences emerged either in the expression of the key enzymes of the endocannabinoid pathway, DAGL-alfa (Mann-Whitney U test, *p* = 0.232), and DAGL-beta (Mann-Whitney U test, *p* = 0.081, Fig. 3A), or of the dopamine receptors DRD1 (Mann-Whitney U test, *p* = 0.527, Supplementary Fig. 1A), DRD2 (Mann-Whitney U test, *p* = 0.798, Supplementary Fig. 1B), or of the dopamine transporter DAT, (Mann-Whitney U test, *p* = 0.945, Supplementary Fig. 1C). The increase of the mRNA levels of CB1 receptor was selective for the mPFC, as no difference was found in NAc, CPu, and amygdala (Supplementary Fig. 2). Given the observed selective increase in CB1 receptor mRNA levels in the mPFC, we conducted further investigations to determine whether a similar increase could be observed in the levels of the CB1 protein in this brain region. Surprisingly, our western blot analysis revealed a significant decrease in CB1 protein levels (Student’s *t*-test, *p = 0.040, Fig. 3B) in *Fgf14*^*−/−*^ mice compared to their WT littermates.

### Basal and stress-induced neuronal activation is preserved in *Fgf14*^−/−^ mice

**Fig. 4:**
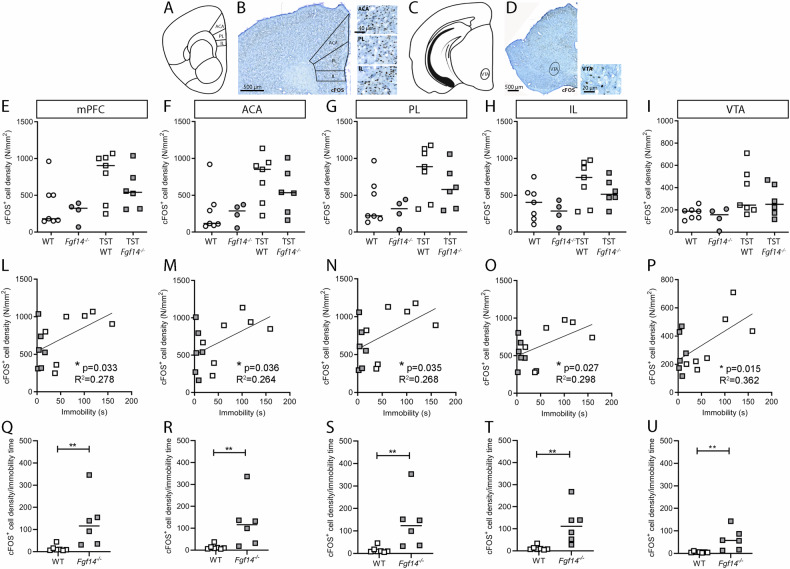
Neuronal activation under basal and following acute stress in the mPFC and VTA. Scheme **A**, **C** and representative images **B**, **D** of the coronal sections of mPFC **A**, **B** and VTA **C**, **D** immunostained with cFOS antibody. cFOS^+^ cell density was evaluated in the whole mPFC **E**, in its subregions anterior cingulate (ACA) **F**, prelimbic (PL) **G** and infralimbic (IL) **H** areas, and VTA **I** of unstressed (circles) and TST-induced acute stress (squares) in WT (empty symbols) and *Fgf14*^*−/−*^ (filled symbols) mice. The analysis reveals that the number of cFOS^+^ cells showed no difference between *Fgf14*^*−/−*^ and WT mice either under basal conditions or following acute stress induced by TST. Positive correlations between immobility time in TST and cFOS^+^ cell density in the whole mPFC **L**, ACA **M**, PL **N** and IL **O** areas, and in VTA **P**. WT: empty squares; *Fgf14*^*−/−*^: filled squares. Ratio of cFOS^+^ cell density over immobility time in whole mPFC **Q**, ACA **R**, PL **S**, IL **T**, and VTA **U**. The analysis reveals that *Fgf14*^*−/−*^ (filled squares) mice show a significantly higher ratio compared to WT (empty squares). The bars indicate median values. *p < 0.05, **p < 0.01.

Neuronal activity was assessed analyzing the expression of the immediate early gene cFOS [46] (Fig. 4A–D and Supplementary Fig. 3). In all brain regions analyzed, the number of cFOS^+^ neurons showed no difference between *Fgf14*^*−/−*^ and WT mice either under basal conditions or following acute stress induced by TST (Fig. 4E–I; Supplementary Fig. 4; Table 1). Since the immobility time was significantly different in *Fgf14*^*−/−*^ compared to WT mice, we evaluated a possible correlation between immobility time and neuronal activity. A positive correlation would indicate that longer immobility times are associated to higher neuronal activation. Considering together the data of *Fgf14*^*−/−*^ and WT mice, a significant positive correlation between cFOS^+^ cell density and the immobility time during TST was detected in multiple brain regions: in all areas of mPFC together (Pearson correlation *p < 0.05, Fig. 4L), in its sub-regions ACA (Pearson correlation *p < 0.05, Fig. 4M), PL (Pearson correlation *p < 0.05, Fig. 4N), and IL (Pearson correlation *p < 0.05, Fig. 4O), and in the VTA (Pearson correlation *p < 0.05, Fig. 4P). No significant positive correlation is present in any other analyzed area, such as the NAc core (Pearson correlation *p* > 0.05, Supplementary Fig. 5A), NAc shell (Pearson correlation *p* > 0.05, Supplementary Fig. 5B), amygdala (Pearson correlation *p* > 0.05, Supplementary Fig. 5C), lateral habenula (Pearson correlation *p* > 0.05, Supplementary Fig. 4D), dorsal HIP (Pearson correlation *p* > 0.05, Supplementary Fig. 5E), and ventral HIP (Pearson correlation *p* > 0.05, Supplementary Fig. 5F). Moreover, we analyzed the correlation between immobility time and neuronal activity in WT and *Fgf14*^*−/−*^ mice separately (Supplementary Fig. 6, Supplementary Table 1).

**Table 1:** Number of cFOS^+^ neurons in different brain regions analyzed, for both *Fgf14*^−/−^ and WT mice either under basal conditions or following acute stress induced by TST.

Area	cFOS+ cell density (N/mm^2^) median (min-max)	Kruskal–Wallis test	Dunn’s post hoc test
mPFC	WT 181,7 (147,2–962,7) TST-WT 903,8 (248,6–1069) *Fgf14*^*−/−*^ 323,8 (69,30–393,7) TST-*Fgf14*^*−/−*^542,0 (309,2–1038)	*p* = 0.051	*p* = 0.093 WT vs TST-WT mice *p* = 0.465 *Fgf14*^*−/−*^ vs TST-*Fgf14*^*−/−*^ *p* > 0.999 WT vs *Fgf14*^*−/−*^ *p* > 0.999 TST-WT vs *TST*-*Fgf14*^*−/−*^
ACA	WT 118,5 (80,94–920,1) TST-WT 851,8 (224,8–1137) *Fgf14*^*−/−*^ 288,5 (57,19–373,5) TST-*Fgf14*^*−/−*^ 535,3 (163,7–1009)	*p = 0.043	*p* = 0.056 WT vs TST-WT mice *p* = 0.617 *Fgf14*^*−/−*^ vs TST-*Fgf14*^*−/−*^ *p* > 0.999 WT vs *Fgf14*^*−/−*^ *p* > 0.999 TST-WT vs *TST*-*Fgf14*^*−/−*^
PL	WT 221,5 (134,6–968,8) TST-WT 888,3 (313,6–1179) *Fgf14*^*−/−*^ 318,3 (34,92–443,7) TST-*Fgf14*^*-/-*^ 579,1 (294,7–1060)	*p* = 0.052	*p* = 0.085 WT vs TST-WT mice *p* = 0.661 *Fgf14*^*−/−*^ vs TST-*Fgf14*^*−/−*^ *p* > 0.999 WT vs *Fgf14*^*−/−*^ *p* > 0.999 TST-WT vs TST-*Fgf14*^*−/−*^
IL	WT 404,2 (103,9–751,5) TST-WT 741,3 (277,9–977,4) *Fgf14*^*−/−*^ 287,2 (57,57–432,5) TST-*Fgf14*^*−/−*^ 514,4 (279,6–804,5)	*p* = 0.052	*p* = 0.235 WT vs TST-WT mice; *p* = 0.283 *Fgf14*^*−/−*^ vs TST- *Fgf14*^*−/−*^ *p* > 0.999 WT vs *Fgf14*^*−/−*^ *p* > 0.999 TST-WT vs *TST*-*Fgf14*^*−/−*^
VTA	WT 189,1 (104,1–258,6) TST-WT 243,9 (161,4–710,4) *Fgf14*^*−/−*^ 156,9 (10,43–209,0) TST-*Fgf14*^*−/−*^ 251,6 (116,0–469,9)	*p* = 0.057	*p* = 0.125 WT vs TST-WT mice; *p* = 0.417 *Fgf14*^*−/−*^ vs TST-*Fgf14*^*−/−*^ *p* > 0.999 WT vs *Fgf14*^*−/−*^ *p* > 0.999 TST-WT vs *TST*-*Fgf14*^*−/−*^
NAc core	WT 105,4 (37,98–506,6) TST-WT 439,8 (77,67–533,5) *Fgf14*^*−/−*^ 54,61 (5,150–107,0) TST-*Fgf14*^*−/−*^ 232,8 (26,17–519,0)	*p* = 0.053	*p* = 0.522 WT vs TST-WT mice; *p* = 0.194 *Fgf14*^*−/−*^ vs TST- *Fgf14*^*−/−*^ *p* = 0.663 WT vs *Fgf14*^*−/−*^ *p* > 0.999 TST-WT vs *TST*-*Fgf14*^*−/−*^
NAc shell	WT 134,5 (47,39–411,9) TST-WT 382,4 (65,79–587,7) *Fgf14*^*−/−*^ 64,07 (4,310–88,93) TST-*Fgf14*^*−/−*^ 245,2 (35,87–507,4)	*p* = 0.063	*p* = 0.522 WT vs TST-WT mice; *p* = 0.194 *Fgf14*^*−/−*^ vs TST- *Fgf14*^*−/−*^ *p* = 0.812 WT vs *Fgf14*^*−/−*^ *p* > 0.999 TST-WT vs TST-*Fgf14*^*−/−*^
Amygdala	WT 235,8 (93,21–346,7) TST-WT 320,0 (135,2–516,1) *Fgf14*^*−/−*^ 121,3 (25,69–224,1) TST-*Fgf14*^*−/−*^ 225,4 (122,7–412,7)	*p* = 0.083	*p* = 0.648 WT vs TST-WT mice; *p* = 0.319 *Fgf14*^*−/−*^ vs TST- *Fgf14*^*−/−*^ *p* = 0.724 WT vs *Fgf14*^*−/−*^ *p* > 0.999 TST-WT vs TST-*Fgf14*^*−/−*^
Habenula	WT 146,2 (37,37–313,4) TST-WT 227,7 (77,49–357,6) *Fgf14*^*−/−*^ 70,01 (38,04–119,7) TST-*Fgf14*^*−/−*^ 161,9 (71,12–431,0)	*p* = 0.095	*p* = 0.795 WT vs TST-WT mice; *p* = 0.294 *Fgf14*^*−/−*^ vs TST- *Fgf14*^*−/−*^ *p* = 0.683 WT vs *Fgf14*^*−/−*^ p > 0.999 TST-WT vs TST-*Fgf14*^*−/−*^
dorsal HIP	WT 71,68 (43,01–184,3) TST-WT 140,9 (67,27–211,4) *Fgf14*^*−/−*^ 65,07 (17,28–80,02) TST-*Fgf14*^*−/−*^ 125,9 (56,49–329,2)	*p* = 0.101	*p* = 0.522 WT vs TST-WT mice; *p* = 0.240 *Fgf14*^*−/−*^ vs TST- *Fgf14*^*−/−*^ *p* > 0.999 WT vs *Fgf14*^*−/−*^ *p* > 0.999 TST-WT vs TST-*Fgf14*^*−/−*^
ventral HIP	WT 83,88 (47,47–183,0) TST-WT 150,1 (100,8–228,5) *Fgf14*^*−/−*^ 70,05 (8,731–103,8) TST-*Fgf14*^*−/−*^ 154,6 (101,2–318,3)	**p = 0.0073	*p* = 0.180 WT vs TST-WT mice; *p = 0.019 *Fgf14*^*−/−*^ vs TST- *Fgf14*^*−/−*^ *p* > 0.999 WT vs *Fgf14*^*−/−*^ *p* > 0.999 TST-WT vs TST-*Fgf14*^*−/−*^

Medial prefrontal cortex mPFC, anterior cingulate area ACA, prelimbic area PL, infralimbic area IL, ventral tegmental area VTA; nucleus accumbens NAc, hippocampus HIP. *p < 0.05, **p < 0.01

Considering the obtained positive correlation in the medial prefrontal cortical areas when WT e *Fgf14*^*−/−*^ mice are considered together, the cFOS^+^ cell density was normalized with the immobility time during TST to obtain a ratio. Notably, *Fgf14*^*−/−*^ mice showed a significant increase in such ratio in comparison to WT mice in all mPFC areas (Mann-Whitney U test, **p = 0.005, Fig. 4Q), in the ACA (Mann-Whitney U test, **p = 0.005, Fig. 4R), in the PL (Mann-Whitney U test, **p = 0.005, Fig. 4S), in the IL (Mann-Whitney U test, **p = 0.002, Fig. 4T), and in the VTA (Mann-Whitney U test, **p = 0.001, Fig. 4U).

### TST-induced activation of GABA and non-GABAergic cells in the mPFC

**Fig. 5:**
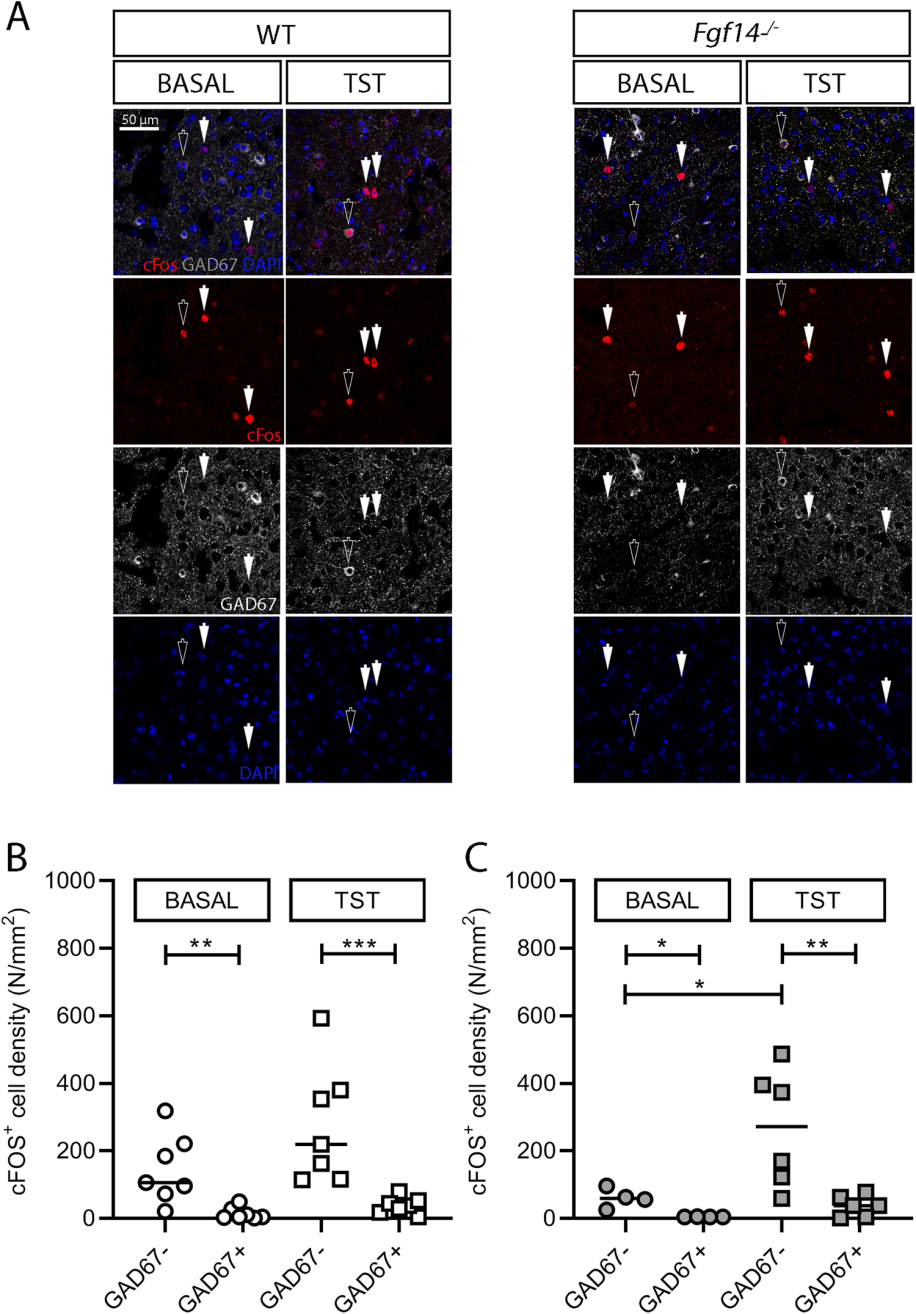
Neuronal activation of GABAergic and non-GABAergic cells in the mPFC. **A** Representative images of the coronal sections of mPFC of WT and *Fgf14*^*−/−*^ mice under basal conditions or following acute stress induced by TST immunostained with cFOS (red) and GAD67 (white) antibody. cFOS^+^ cell density was evaluated in the non-GABAergic (GAD67^-^) and GABAergic (GAD67^+^) cells of whole mPFC in WT **B** and *Fgf14*^*−/−*^ mice **C** under basal conditions or following acute stress induced by TST. The analysis reveals that the majority of cFOS^+^ cells is non-GABAergic, and that the amount of such cells is significantly higher after TST only in *Fgf14*^*−/−*^ mice. ^*^p < 0.05, ^**^p < 0.01, ^***^p < 0.001.

Next, we asked whether TST-induced neuronal activation had to be attributed to excitatory or inhibitory neurons, especially in the mPFC where the immobility time is correlated with cFOS expression. GABAergic neurons were detected by GAD67 immunoreactivity (Fig. 5A). The results revealed that, among cFOS^+^ cells, the majority is non-GABAergic (Mann-Whitney U test, cFOS^+^GAD67^+^ vs cFOS^+^GAD67^-^ cells density for WT **p = 0.002; TST-WT ***p = 0.0006; *Fgf14*^*−/−−/−*^ *p = 0.029; TST-*Fgf14*^*−/−*^ **p = 0.009; Fig. 5B, C), indicating that in mPFC, neuronal activation is mainly ascribable to non-GABAergic neurons.

The density of cFOS^+^GAD67^-^ cells was significantly increased after TST only in *Fgf14*^*−/−*^ mice (Kruskal–Wallis test *p* = 0.026 with Dunn’s post hoc test *p* = 0.503 WT vs TST-WT mice; *p = 0.048 Fgf14^*−/−*^ vs TST-Fgf14^*−/−*^, Fig. 5B, C). This result suggests that the resilience to stress of *Fgf14*^*−/−*^ mice is accompanied by a significant increase of activity of pyramidal neurons.

## Discussion

The present work demonstrates that, in female mice, deletion of *Fgf14* induces a reduced level of despair behavior, *anhedonia* and increased sociability. These behavioral changes indicate resilience to depression. A reduction of anxious-like behavior was associated with this resilience to depression. *Fgf14*^*−/−*^ mice display ataxic motor deficits [26]. However, it is highly unlikely that the motor deficits of *Fgf14*^*−/−*^ mice might affect the results of TST, sucrose preference, sociability test or anxiety tests, because it has been reported that the distance travelled by spontaneous walking locomotion and swimming locomotion is not reduced, compared to WT controls [47]. In the EPM and OF tests we confirm that the total distance travelled by *Fgf14*^*−/−*^ mice is not affected by their ataxia. This *Fgf14* phenotype is in line with the fact that GSK3, which is a key element of *Fgf14* modulation, is also involved in depression [36–38]. Under physiological conditions, active GSK3 phosphorylates *Fgf14* and Na_v_, stabilizing the correct formation of their complex in the AIS to maintain neuronal excitability [48]. Following the pharmacological inhibition of GSK3, the formation of the *Fgf14*-Na_v_ channel complex is reduced [35]. Consequently, our depression-resilient *Fgf14*^*−/−*^ mouse model could be partially assimilated to a condition in which GSK3 is inactive, and therefore *Fgf14* is no longer able to control the correct localization in the AIS of Na_v_. Pharmacological inhibition of GSK3 has antidepressant effects in animal models of depression so that GSK3 inhibitors could potentially be used as antidepressants [49]. In line with the antidepressant action of GSK3 inhibitors, our results show that *Fgf14* deletion also has an antidepressant effect. This suggests that the antidepressant effect of GSK3 might by mediated, at least in part, by its action on *Fgf14*.

Since endocannabinoid and dopaminergic neurotransmission is involved in the molecular mechanisms underlying depression [8, 9], we analyzed some key molecules of these two systems in the mPFC, which is an important hub of the network involved in mood disorders [2–4]. Regarding CB1 receptors, we found reduced protein levels in *Fgf14*^*−/−*^ mice compared to WT. It is noteworthy that most of the available treatments for depression have been observed to modulate endocannabinoid signaling. Indeed, the therapeutic effects of antidepressants can be attributed to their ability to alter the number of CB1 receptors, the synthesis of anandamide, or the interaction between endocannabinoids and CB1 receptors [50–52]. The results obtained by multiple studies are contradictory. However, when considered collectively, these findings support the hypothesis that the recruitment of the endocannabinoid system may be involved in the aetiology of depression. On the other hand, our results demonstrate that, in the mPFC of *Fgf14*^*−/−*^ mice, there are no changes in the expression of dopamine receptors or dopamine transporter. This finding is in agreement with a previous report showing intact dopamine levels in *Fgf14*^*−/−*^ mice [26].

Stress-induced depression- and anxiety-like behaviors in murine models have been associated with a strong cFOS induction in many brain regions, including the mPFC [53]. Therefore, we analyzed cFOS expression as a marker of neuronal activity under basal conditions and following TST, in WT and *Fgf14*^*−/−*^ mice. Immobility time in the TST was highly variable in WT mice, ranging from 18–159 s. We reasoned that the degree of neuronal activation assessed by cFOS expression could be correlated to the severity of despair behavior, measured by the immobility time. These two parameters were significantly correlated, with higher cFOS+ cell density corresponding to longer immobility times. Such correlation was found specifically in the mPFC and VTA, suggesting that these might be the critical regions involved in resilience to depression of *Fgf14*^*−/−*^ mice. This finding agrees with the widely accepted concept that the mPFC is a crucial area implicated in the responses to stress and depression [2, 3]. Next, we asked whether *Fgf14*^*−/−*^ mice had a density of cFOS+ cells in line with the correlation found in WT controls. *Fgf14*^*−/−*^ mice showed much less immobility than WT mice, but, following such correlation, the low immobility time of *Fgf14*^*−/−*^ mice was expected to correspond to low counts of cFOS+ cells. Interestingly, in contrast to this expectation, *Fgf14*^*−/−*^ mice showed a significantly higher ratio between cFOS expression and the immobility time, suggesting that such enhanced neuronal activation might contribute to the mechanism of resilience to depression. Our results also demonstrate that most of the activated neurons are mainly pyramidal neurons in mPFC. In *Fgf14*^*−/−*^ mice we find that, after TST-induced acute stress, there is a significant activation only of pyramidal neurons. *Fgf14* is localized to the axon initial segment, which is the critical cellular compartment where action potentials are triggered. For this reason, a future development of this study is understanding the role of *Fgf14* in the generation of action potentials by mPFC pyramidal neurons after TST-induced acute stress.

On one hand the chronic activation of excitatory pyramidal neurons of the mPFC in rats has been found to induce depression-related behaviors like decreased sociability and reduced *anhedonia* [54]. On the other hand, short-term stimulation in mice of pyramidal neurons of the mPFC at a slow, physiological, frequency was found to synchronize the activity of NAc, amygdala and VTA, resulting in antidepressant effects [55]. Our results of a higher activation of pyramidal neurons after TST in Fgf14^*−/−*^ mice cannot distinguish between different patterns of action potential firing, and in principle might be either pro- or anti-depressant. However, resilience to depression of *Fgf14*^*−/−*^ mice might be linked to a type of neuronal activity which is able to synchronize mPFC targets. Future experiments are required to detect the activity pattern of pyramidal neurons and the synchronization of the stress-related circuit. Our results suggest that *Fgf14* is involved in the stress-coping mechanisms and be targeted to improve the resilience to depression.

## Supplementary information

Figure legends

Suppl Figure 1

Suppl Figure 2

Suppl Figure 3

Suppl Figure 4

Suppl Figure 5

Suppl Figure 6

Suppl Table 1

## Data Availability

All data will be made available upon reasonable request to the corresponding author.
