# Supplementary material for: Deletion of Fgf14 confers resilience to basal and stress-induced depressive-like behavior and reduces anxiety in mice
Source: Transl Psychiatry. 2025 Apr 9;15:136. doi: 10.1038/s41398-025-03361-z (PMC11982207; doi:10.1038/s41398-025-03361-z)
Supplement: Supplementary file 1 — Figure legends [file 41398_2025_3361_MOESM1_ESM.docx]

**Supplementary figure 1.** **Expression levels of the dopaminergic system in the mPFC.** Comparison of gene expression levels in the mPFC of WT (empty circles) and *Fgf14^-/-^* (filled circles) mice of dopamine receptor 1 (DRD1) (A), dopamine receptor 2 (DRD2) (B), dopamine transporter (DAT) (C). *Fgf14^-/-^* mice show comparable gene expression level for all the analyzed genes compared to WT. The bars indicate median values.

**Supplementary figure** 2. **Expression levels of cannabinoid receptor 1 (CB1).** Comparison of gene expression levels of CB1 in the nucleus accumbens (NAc, A), caudate putamen (CPu, B) and amygdala (C) of WT (empty circles) and *Fgf14^-/-^* (filled circles). Expression levels are not different between genotypes.

**Supplementary figure 3.** Representative images of the coronal sections of mPFC (A, G, M, S), ACA (B, H, N, T), PL (C, I, O, U), IL (D, J, P, V) and VTA (E, F, K, L, Q, R, W, X) of unstressed (A-L) and TST-induced acute stressed (M-X) WT (A-F, M-R) and Fgf14-/- (G-L, S-X) mice, immunostained with cFOS antibody.

**Supplementary figure 4. Quantitative analysis of cFOS immunostaining of WT and *Fgf14^-/-^* mice in other brain areas involved in depression.** cFOS^+^ cell density was evaluated in the nucleus accumbens (NAc) core (A), NAc shell (B), amygdala (C), lateral habenula (D), dorsal hippocampus (HIP) (E), and ventral HIP (F) of unstressed (circles) and TST-induced acute stress (squares). Empty symbols: WT mice; filled symbols: *Fgf14^-/-^* mice. The analysis reveals that *Fgf14^-/-^* mice subjected to TST show a significantly higher number of cFOS^+^ cells compared to unstressed controls only in ventral HIP (F). *p < 0.05.

**Supplementary figure 5. Correlation analysis between cFOS^+^ cell density and immobility time after TST-induced acute stress in other brain areas involved in depression.** Correlation between immobility time in TST and cFOS^+^ cell density in the NAc core (A) NAc shell (B), amygdala (C), lateral habenula (D), dorsal HIP (E), and ventral HIP (F). Empty squares: WT mice; filled squared: *Fgf14^-/-^* mice. The analysis reveals no correlations between immobility time in TST and cFOS^+^ cell density in all the analysed regions. *p < 0.05.

**Supplementary figure 6. Correlation analysis between cFOS^+^ cell density and immobility time after TST-induced acute stress in WT mice for all brain areas involved in depression.** Correlations between immobility time in TST and cFOS^+^ cell density in the whole mPFC (A), ACA (B), PL (C), IL (D) areas, VTA (E), NAc core (F), NAc shell (G), amygdala (G), lateral habenula (H), dorsal HIP (I), and ventral HIP (J) of WT mice. *p < 0.05.
