# Supplementary figures and images for: Deletion of Fgf14 confers resilience to basal and stress-induced depressive-like behavior and reduces anxiety in mice
Source: Transl Psychiatry. 2025 Apr 9;15:136. doi: 10.1038/s41398-025-03361-z (PMC11982207; doi:10.1038/s41398-025-03361-z)

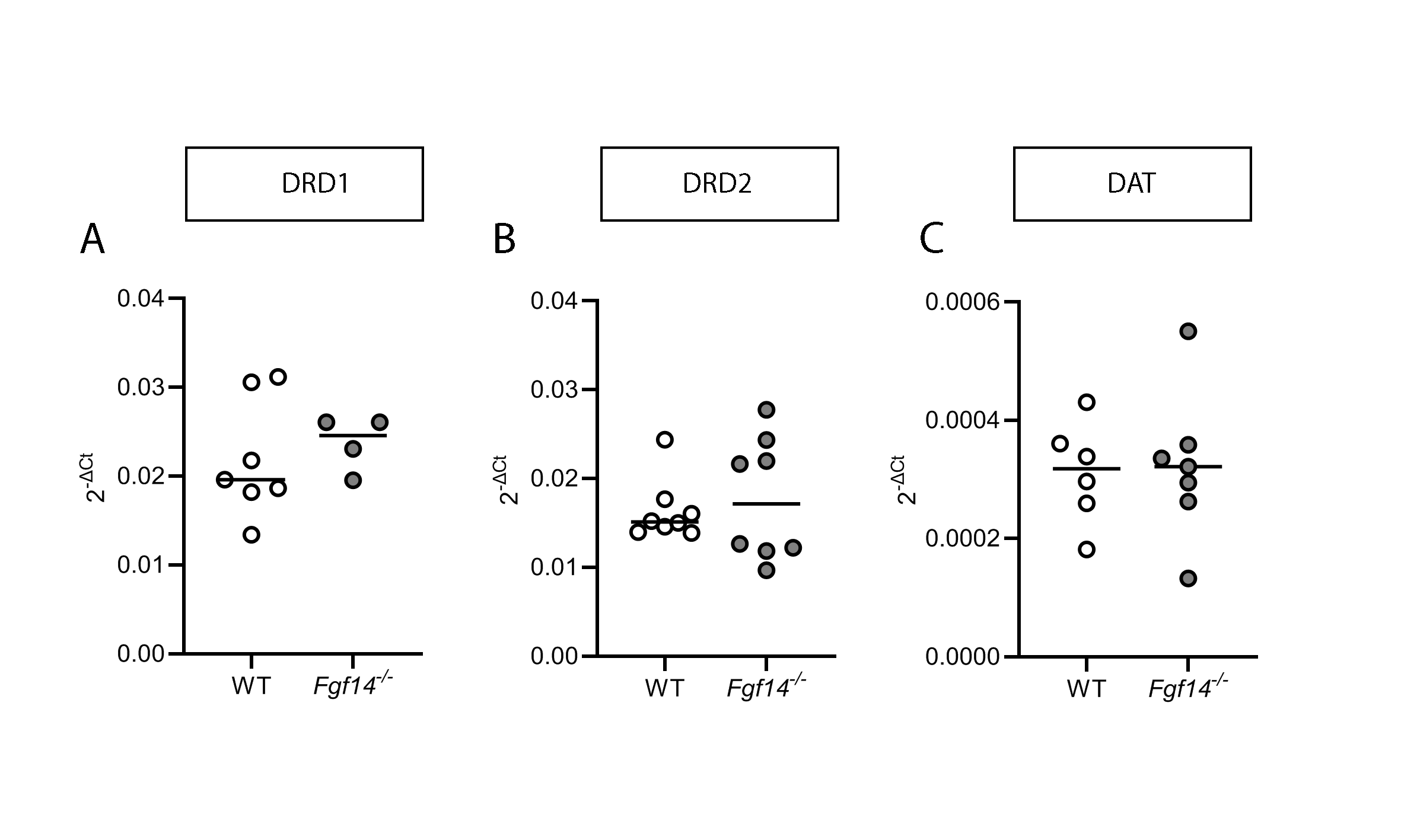

Supplement: Supplementary file 2 — Suppl Figure 1 [file 41398_2025_3361_MOESM2_ESM.tif]

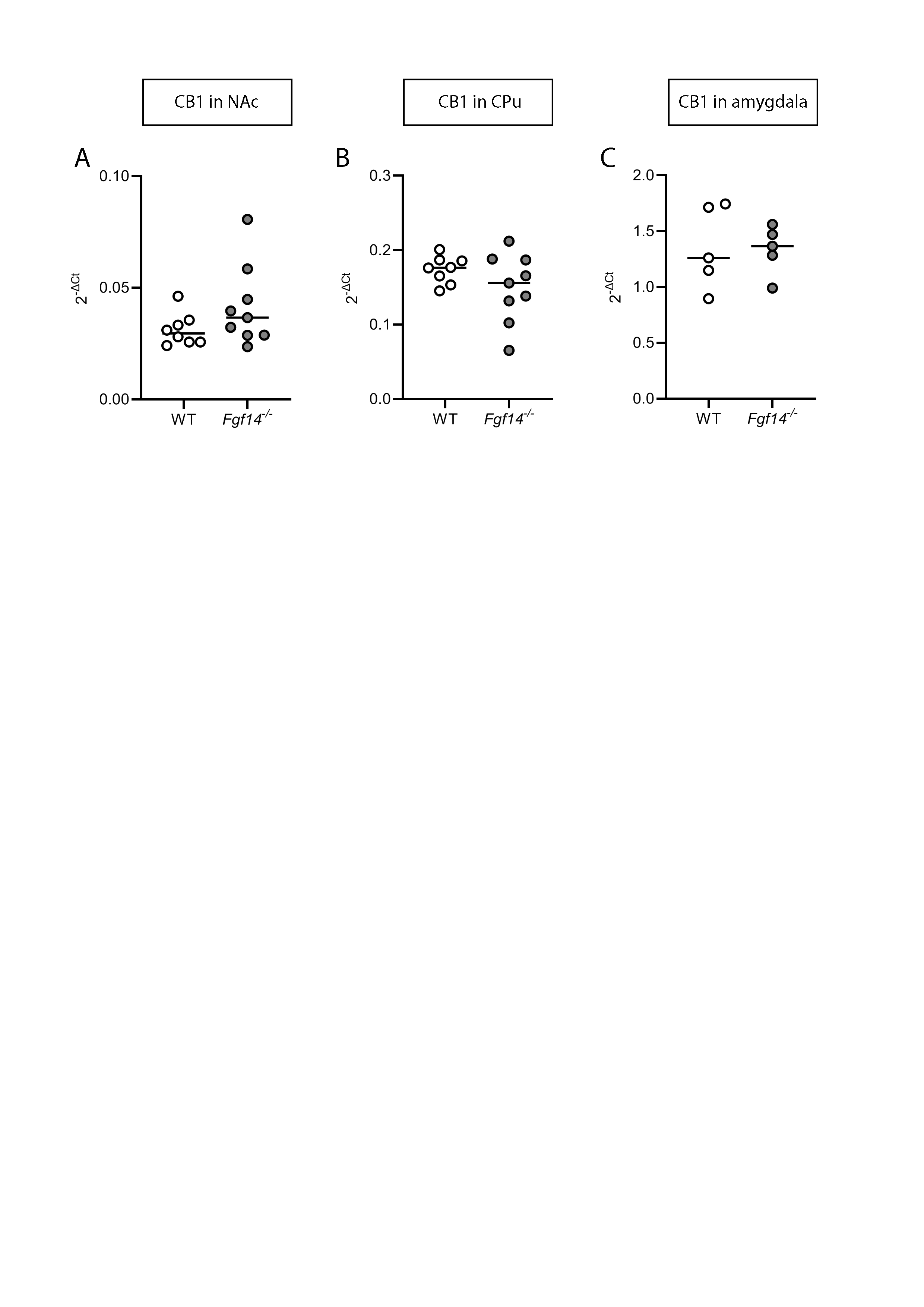

Supplement: Supplementary file 3 — Suppl Figure 2 [file 41398_2025_3361_MOESM3_ESM.tif]

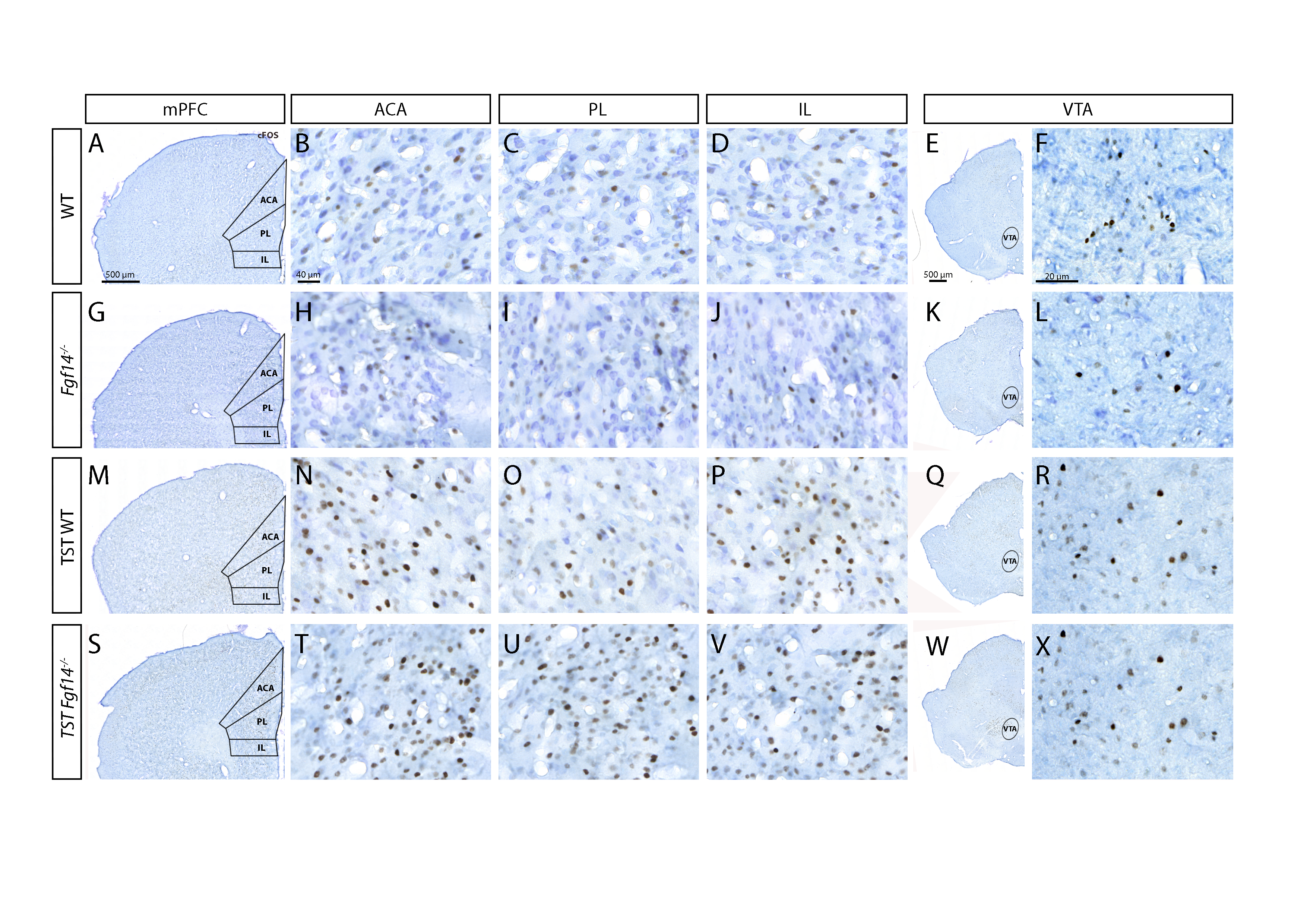

Supplement: Supplementary file 4 — Suppl Figure 3 [file 41398_2025_3361_MOESM4_ESM.tif]

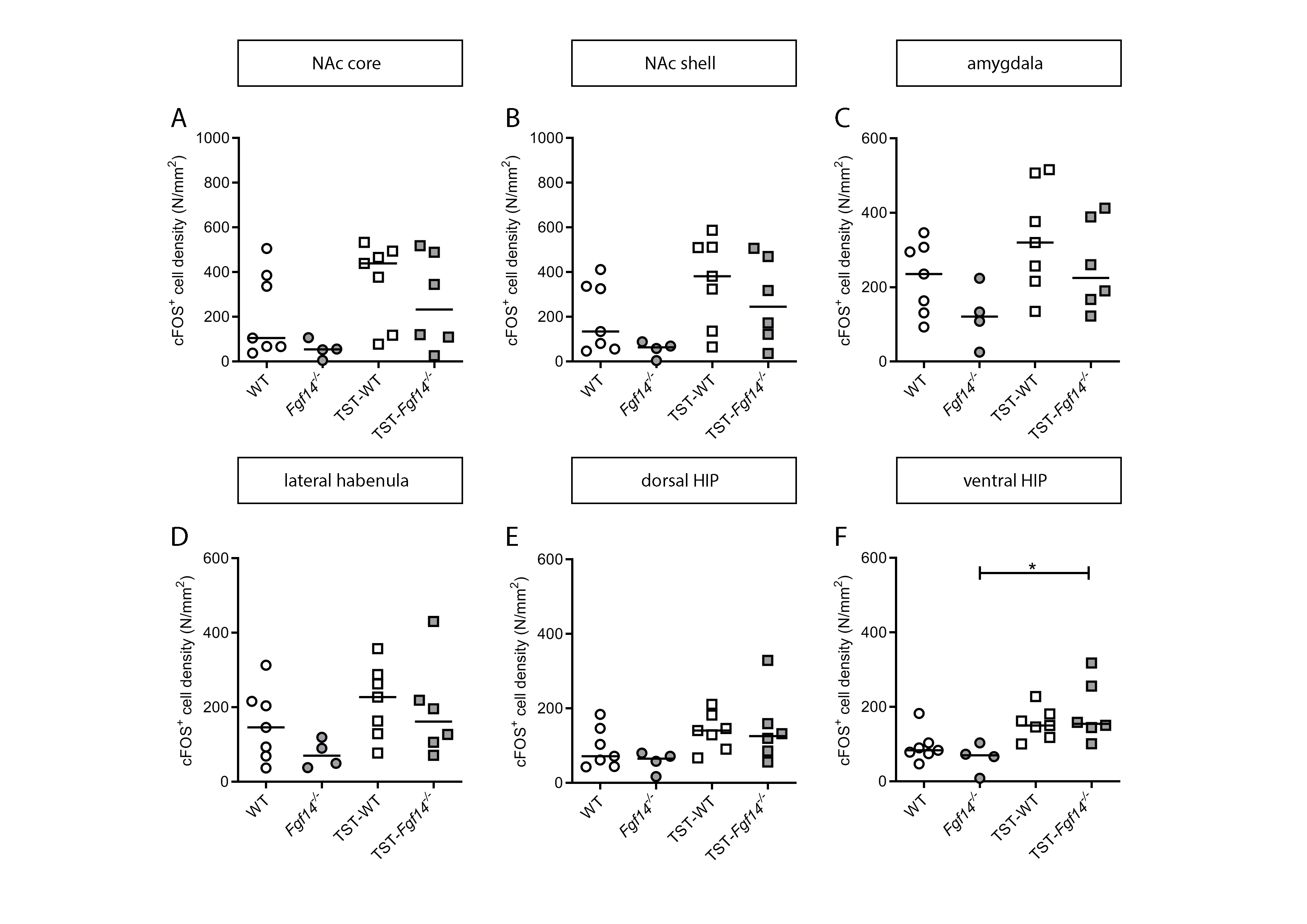

Supplement: Supplementary file 5 — Suppl Figure 4 [file 41398_2025_3361_MOESM5_ESM.tif]

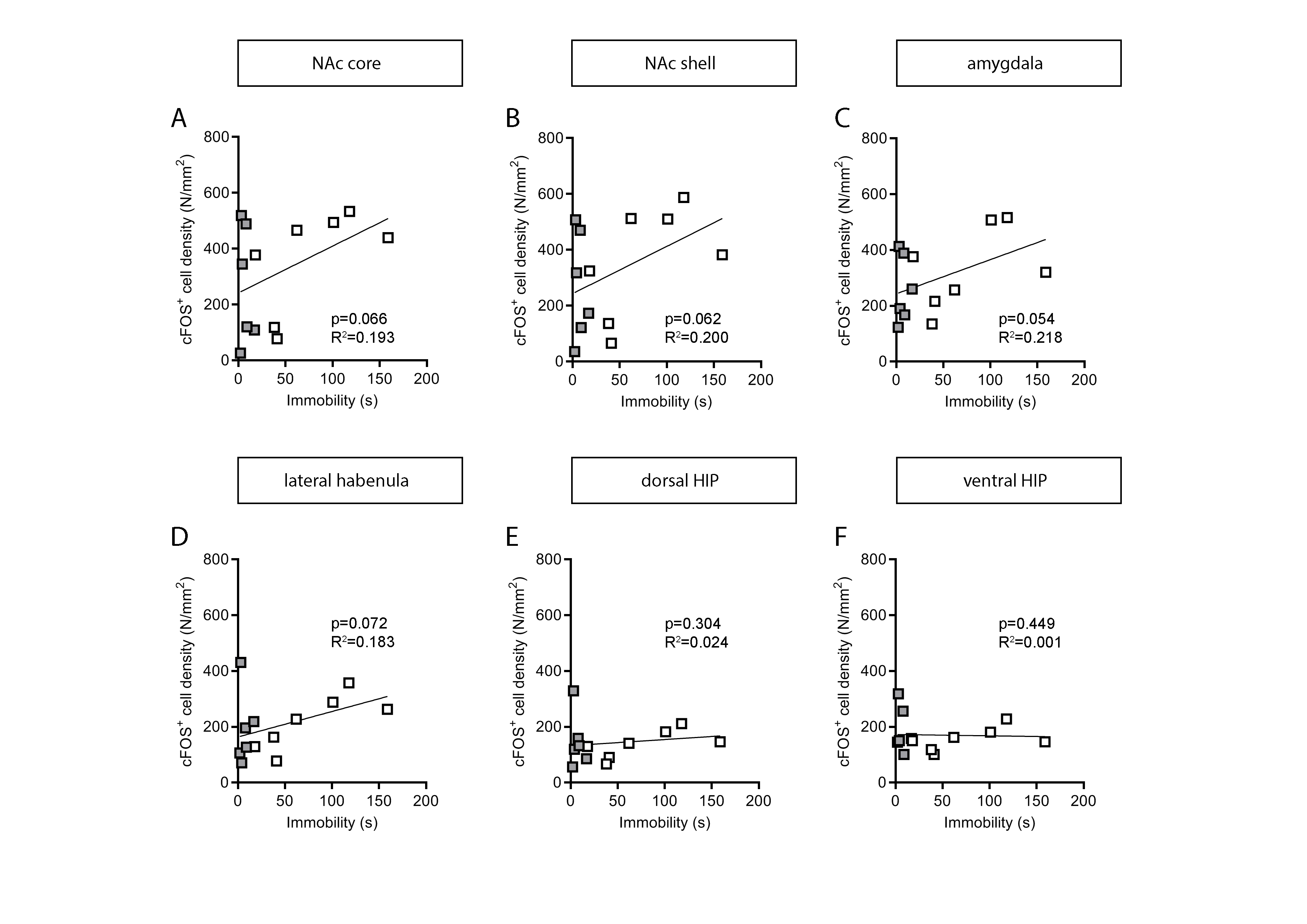

Supplement: Supplementary file 6 — Suppl Figure 5 [file 41398_2025_3361_MOESM6_ESM.tif]

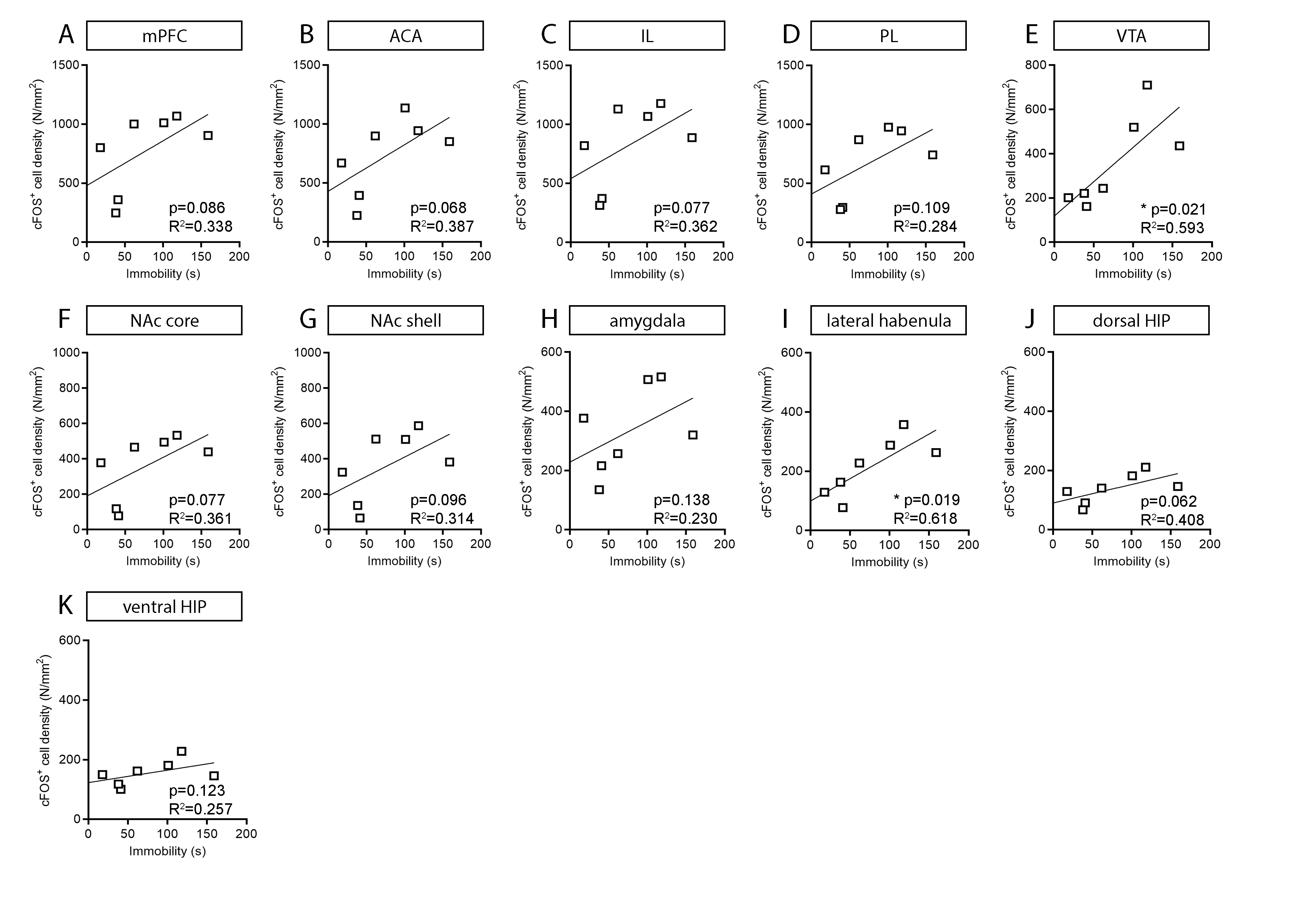

Supplement: Supplementary file 7 — Suppl Figure 6 [file 41398_2025_3361_MOESM7_ESM.tif]
