# Supplementary material for: Deletion of Fgf14 confers resilience to basal and stress-induced depressive-like behavior and reduces anxiety in mice
Source: Transl Psychiatry. 2025 Apr 9;15:136. doi: 10.1038/s41398-025-03361-z (PMC11982207; doi:10.1038/s41398-025-03361-z)
Supplement: Supplementary file 8 — Suppl Table 1 [file 41398_2025_3361_MOESM8_ESM.docx]

| **Area** | **WT mice (n=7)** | ***Fgf14^-/-^* mice (n=6)** | **Total mice (n=13)** |
| --- | --- | --- | --- |
| **mPFC** | p=0.086  R^2^=0.338 | p=0.745  R^2^=0.029 | * p=0.033  R^2^=0.278 |
| **ACA** | p=0.068  R^2^=0.387 | p=0.829  R^2^=0.013 | * p=0.036  R^2^=0.264 |
| **PL** | p=0.077  R^2^=0.362 | p=0.779  R^2^=0.022 | * p=0.035  R^2^=0.268 |
| **IL** | p=0.109  R^2^=0.284 | p=0.847  R^2^=0.010 | * p=0.027  R^2^=0.298 |
| **VTA** | * p=0.021  R^2^=0.593 | p=0.977  R^2^=0.0002 | * p=0.015  R^2^=0.362 |
| **NAc core** | p=0.077  R^2^=0.361 | p=0.581  R^2^=0.082 | p=0.066  R^2^=0.193 |
| **NAc shell** | p=0.096  R^2^=0.314 | p=0.725  R^2^=0.034 | p=0.062  R^2^=0.200 |
| **amygdala** | p=0.138  R^2^=0.230 | p=0.893  R^2^=0.006 | p=0.054  R^2^=0.218 |
| **lateral habenula** | * p=0.019  R^2^=0.618 | p=0.993  R^2^=0.00002 | p=0.072  R^2^=0.183 |
| **dorsal HIP** | p=0.062  R^2^=0.408 | p=0.552  R^2^=0.095 | p=0.304  R^2^=0.024 |
| **ventral HIP** | p=0.123  R^2^=0.257 | p=0.617  R^2^=0.068 | p=0.449  R^2^=0.001 |

**Supplementary table 1.** Pearson’s correlation between cFOS+ cell density and immobility time after TST-induced acute stress
